# Increased Causal Connectivity Related to Anatomical Alterations as Potential Endophenotypes for Schizophrenia

**DOI:** 10.1097/MD.0000000000001493

**Published:** 2015-10-23

**Authors:** Wenbin Guo, Feng Liu, Changqing Xiao, Miaoyu Yu, Zhikun Zhang, Jianrong Liu, Jian Zhang, Jingping Zhao

**Affiliations:** From the Mental Health Institute of the Second Xiangya Hospital, Key Laboratory of Psychiatry and Mental Health of Hunan Province, Central South University, Changsha, Hunan (GW, ZJ); Key Laboratory for NeuroInformation of Ministry of Education, School of Life Science and Technology, University of Electronic Science and Technology of China, Chengdu, Sichuan (LF); and Mental Health Center, The First Affiliated Hospital, Guangxi Medical University; Nanning, Guangxi, China (XC, YM, ZZ, LJ, ZJ).

## Abstract

Anatomical and functional abnormalities in the cortico-cerebellar-thalamo-cortical circuit have been observed in schizophrenia patients and their unaffected siblings. However, it remains unclear to the relationship between anatomical and functional abnormalities within this circuit in schizophrenia patients and their unaffected siblings, which may serve as potential endophenotypes for schizophrenia.

Anatomical and resting-state functional magnetic resonance imaging data were acquired from 49 first-episode, drug-naive schizophrenia patients, 46 unaffected siblings, and 46 healthy controls. Data were analyzed by using voxel-based morphometry and Granger causality analysis.

The patients and the siblings shared anatomical deficits in the left middle temporal gyrus (MTG) and increased left MTG–left angular gyrus (AG) connectivity. Moreover, the left MTG–left AG connectivity negatively correlates to the duration of untreated psychosis in the patients.

The findings indicate that anatomical deficits in the left MTG and its increased causal connectivity with the left AG may serve as potential endophenotypes for schizophrenia with clinical implications.

## INTRODUCTION

Schizophrenia is a serious and highly heritable psychiatric disorder with a lifetime incidence of 1% in general population.^1^ This chronic disease is characterized by symptoms of hallucinations, delusions, affective apathy, and cognitive deficits.^2^ Although the exact neurobiology of schizophrenia remains unclear, the “disconnection” hypothesis has been proposed that schizophrenia may be the result of disruptive integration of massive brain regions comprising brain circuits by neuroimaging studies,^3,4^ such as the default-mode network^5^ and cortico-cerebellar-thalamo-cortical circuit (CCTCC).^6^

Among these circuits, the CCTCC is one of the most examined circuits, which is a crucial player in the neurobiology of schizophrenia.^7,8^ Both anatomical and functional abnormalities of brain regions within this circuit have been revealed by neuroimaging studies. For example, gray matter volume reduction of this circuit has been observed in patients with schizophrenia, including brain regions such as the anterior cingulate cortex, frontal and temporal cortices, amygdala, thalamus, hippocampus, parahippocampal gyrus, and insula.^9,10^ Disrupted white matter connectivity of this circuit has been found in patients by diffusion tensor imaging studies, such as decreased fractional anisotropy in the internal capsule, external capsule, fornix, and superior longitudinal fasciculus.^11–13^ Functional deficits of this circuit, including brain regions in the frontal, temporal, and parietal cortices, have been implicated in the neurobiology of schizophrenia by using resting-state functional magnetic resonance imaging.^14–16^ However, it remains unclear how anatomical deficits are related to functional alterations in schizophrenia.

The above-mentioned studies provide valuable evidence of dysconnectivity in the CCTCC in the neurobiology of schizophrenia. However, most of these studies have recruited chronic and medicated patients, which may introduce numerous confounding factors such as long illness duration and medication use. For example, prolonged illness duration is associated with decreased gray matter volume in the right superior temporal gyrus and caudate nucleus.^17^ Medication use has a treatment-related effect on both anatomical and functional results of neuroimaging studies.^18,19^ Hence, the recruitment of first-episode, drug-naive patients is meaningful.

Unaffected siblings of schizophrenia patients share similar early-life environment and genetic background with the patients, and have a higher risk to develop schizophrenia than the general population.^20^ Moreover, unaffected siblings show similar brain alterations that are also found in the patients,^21–27^ including anatomical abnormalities in the temporal gyrus, thalamus, and amygdala-hippocampal complex,^28,29^ and functional abnormalities in the frontal, temporal, and parietal gyri.^21,22^ Conducting a study with both the patients and their unaffected siblings may be helpful to explore potential endophenotypes for schizophrenia. An endophenotype is heritable and shown to segregate with disease within families.^20^ According to its concept, brain anatomical and functional abnormalities shared by schizophrenia patients and their unaffected siblings may serve as potential endophenotypes for schizophrenia. Previously, we observed that first-episode, drug-naive schizophrenia patients had abnormal causal connectivity in the prefrontal-thalamic (limbic)-cerebellar (sensorimotor) circuit disrupted by structural deficits.^30^ However, it is unclear whether similar abnormalities exist in unaffected siblings, which may serve as potential endophenotypes for schizophrenia.

In the present study, a relatively large sample of schizophrenia patients and their unaffected siblings was recruited to examine abnormal causal connectivity related to anatomical deficits shared by the patients and unaffected siblings, which might serve as potential endophenotypes for schizophrenia. First, we examined anatomical deficits shared by the patients and unaffected siblings using a voxel-based morphometry (VBM) method. Then, brain regions with shared anatomical deficits in the patients and siblings were employed as seeds, and Granger causality analysis (GCA) was used to examine abnormal causal connectivity between the seeds and other brain regions. The aim of this study was to explore the shared anatomical and functional abnormalities in the patients and siblings, which could be regarded as potential endophenotypes for schizophrenia. We also examined the correlations between anatomical and functional abnormalities and clinical variables (i.e., symptom severity).

## MATERIALS AND METHODS

### Participants

This study included 49 first-episode, drug-naive patients, 46 unaffected siblings, and 46 healthy controls. The patients were diagnosed using the Structural Clinical Interview for DSM-IV (SCID), patient edition.^1^ Positive and Negative Symptom Scale was used to rate the symptom severity, and the patients had the duration of untreated psychosis (DUP) of less than 3 years. The siblings had brothers and sisters diagnosed as schizophrenia with SCID, patient edition.^1^ All participants were right-handed, aged from 16 to 30 years with more than 9 years of formal education. The siblings and the controls were screened using SCID, nonpatient edition.^1^ The exclusion criteria for all participants were neurological or psychiatric disorders other than schizophrenia, acute physical disease, substance abuse or dependence, and contraindications for MRI scanning. Healthy controls were excluded if they had a first-degree relative suffering from psychiatric disorders.

The study was conducted in accordance with the Helsinki Declaration,^31^ and the ethics committee of the First Affiliated Hospital, Guangxi Medical University, China approved the study. All participants were informed about the study procedures and gave a written informed consent.

### Scan Acquisition and Data Preprocessing

Anatomical and resting-state functional data were acquired on a Siemens 3T scanner. Preprocessing of anatomical and functional data were carried out using the VBM toolbox (VBM8, http://dbm.neuro.uni-jena.de/vbm) and the DPARSF toolbox,^32^ respectively. Details of scan acquisition and data preprocessing can be found in the online Supplementary file.

### Anatomical Data Analyses

Analyses of variances, followed by post hoc *t*-tests, were conducted to compare the differences of anatomical data between groups. The significance level was set at the corrected *P* < 0.005 level for multiple comparisons using the Gaussian random field (GRF) theory (min z > 2.807, cluster significance: *P* < 0.005). Brain regions with anatomical abnormalities were selected as seeds for GCA analyses.

### GCA Processing

Anatomical results revealed that the patients and the siblings shared anatomical deficits in the left middle temporal gyrus (MTG) compared with the controls. The anatomical automatic labeling of the left MTG was chosen as seed for GCA processing because the clusters with anatomical deficits in the patients and siblings were located in different regions of the left MTG (The reproducibility of this method was checked in the Reproducibility section). Voxel-wise coefficient GCA in the whole brain was performed with the REST Software.^33^ Vector autoregressive models were applied to assess the Granger causality to decide whether the past value of a time series could forecast the present value of another time series correctly. Classic GCA is based on F-test^34,35^ and can only assess the positive effects for the reason that F value is always positive. However, both positive and negative causal effects are important to maintain normal brain function. An improved GCA method, coefficient-based GCA, was proposed to overcome this weakness, and a signed regression coefficient β was used to assess the Granger causality.^30,36–40^ Positive/negative β may represent the excitatory/inhibitory effect or positive/negative feedback.^30,36–40^ In this study, bivariate coefficient GCA was conducted to examine the causal effect between the seed and other voxels of the whole brain. There were 2 analyses: seed-to-whole-brain analysis and whole-brain-to-seed analysis. Finally, coefficient β was z-transformed for standardization purpose.

Since micromovement could affect resting-state FC from volume to volume,^41,42^ we calculated the framewise displacement (FD) values for each participant. The causal effects were compared using analyses of covariances with the mean FD as a covariate, followed by post hoc *t*-tests. The significance level was set at *P* < 0.005 (GRF corrected).

### Correlation Analyses in the Patients

Voxel-based correlations were conducted between anatomical or functional alterations shared by the patients and the siblings and clinical variables, respectively, in the patients with age and sex as covariates (*P* < 0.05, GRF corrected).

## RESULTS

### Characteristics of the Participants

Fourteen patients and 14 siblings are sib pairs. The other participants are from different families and unrelated to each other. The 3 groups have no significant group difference in age, sex ratio, and education level (Table 1). There is also no significant group difference in the mean FD values.

**TABLE 1:**
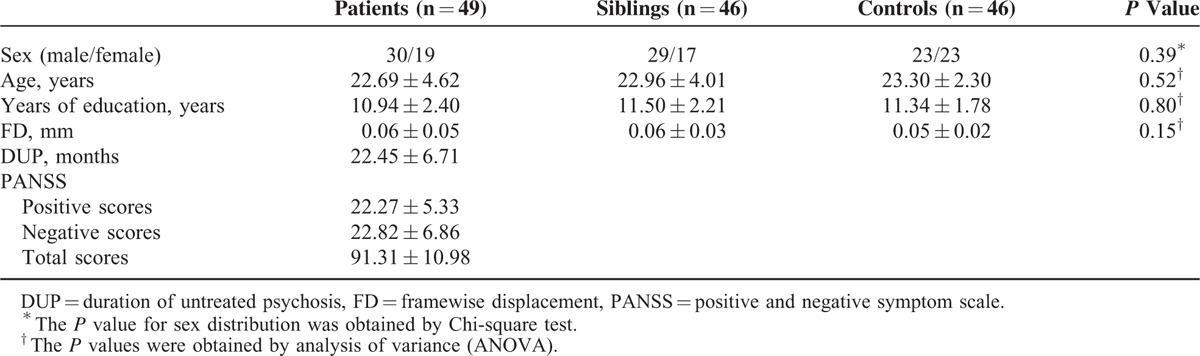
Characteristics of the Participants

### Anatomical Abnormalities Shared by the Patients and the Siblings

**FIGURE 1:**
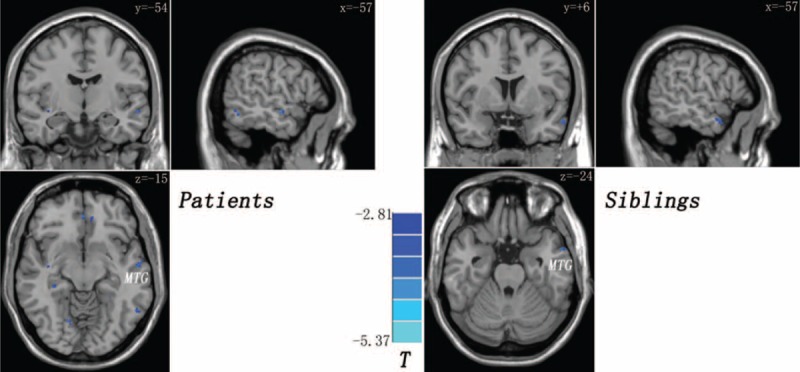
Decreased gray matter volume in the left middle temporal gyrus (MTG) shared by the patients and the siblings (compared with the controls). The color bar indicates the T values from post hoc *t*-tests.

Compared with the controls, the patients and the siblings share anatomical deficits in the left MTG using post hoc *t*-tests (Table 2 and Fig. 1). This brain region is selected as seed for the following GCA analyses.

**TABLE 2:**
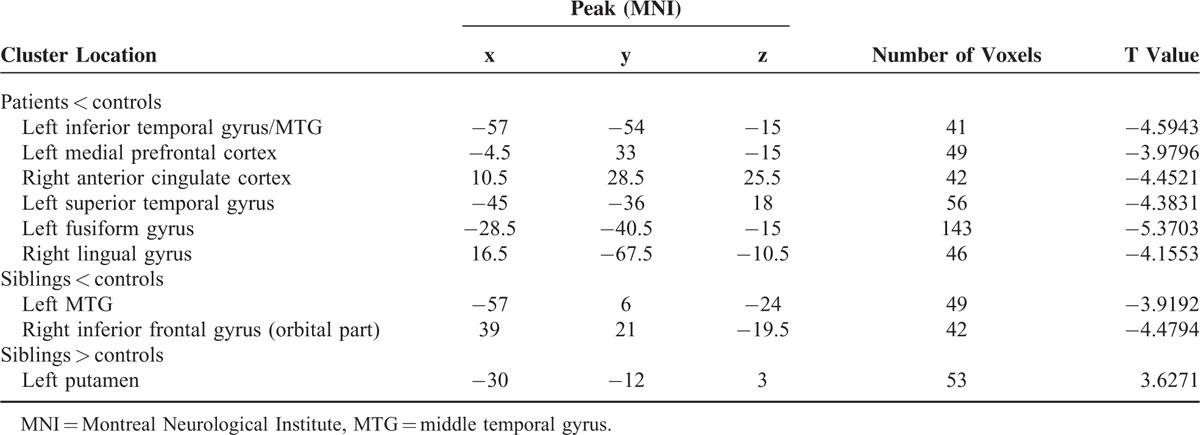
Regions With Abnormal Gray Matter Volume in the Patients and the Siblings

### Voxel-Wise GCA: Seed-to-Whole-Brain Analysis

**FIGURE 2:**
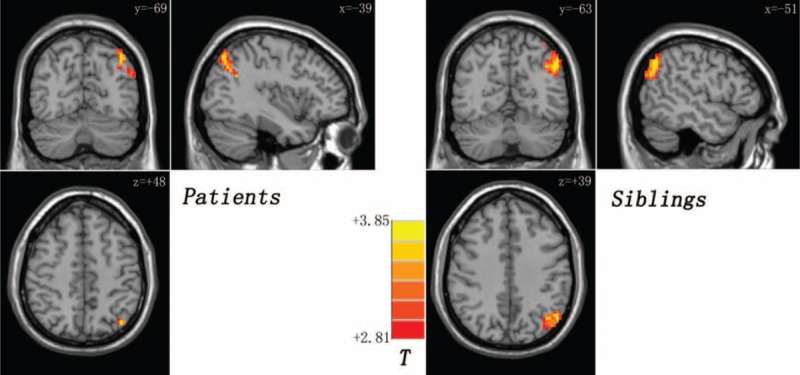
Increased causal connectivity from the left middle temporal gyrus to the left angular gyrus shared by the patients and the siblings (compared with the controls). The color bar indicates the T values from post hoc *t*-tests.

Compared with the controls, the patients and the siblings exhibit excitatory effect from the left MTG to the left angular gyrus (AG) (Table 3 and Fig. 2). The patients show inhibitory effect from the left MTG to the right cerebellum posterior lobe and excitatory effect from the left MTG to the left middle occipital gyrus relative to the siblings (Table 3).

**TABLE 3:**
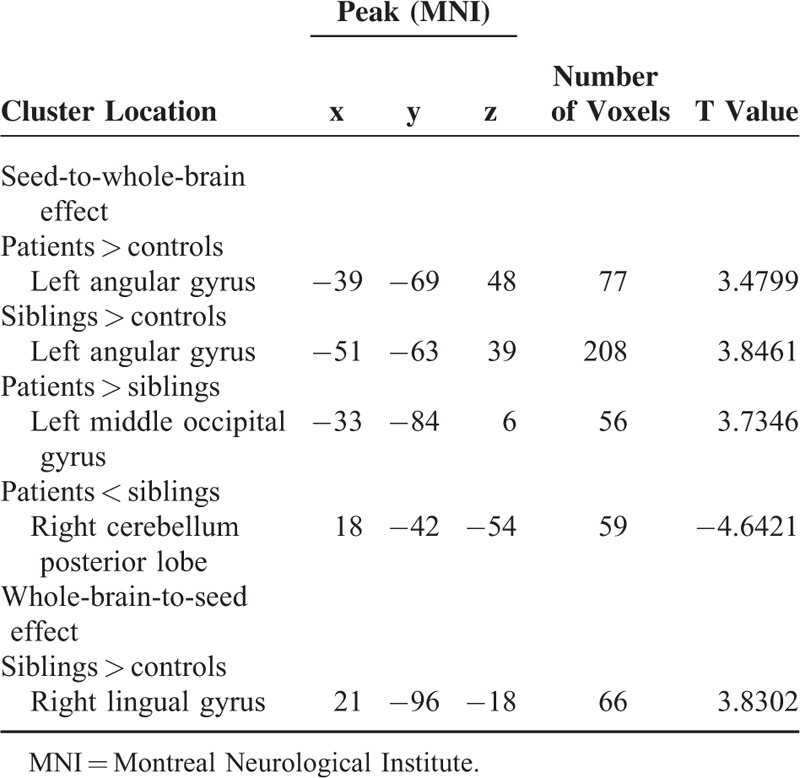
Regions With Abnormal Causal Effect With the Seed (the Anatomical Automatic Labeling of the Left Middle Temporal Gyrus)

### Voxel-Wise GCA: Whole-Brain-to-Seed Analysis

The siblings show positive feedback from the right lingual gyrus to the left MTG compared to the controls (Table 3). No other abnormal feedback is found in the patients and the siblings.

### Reproducibility

**FIGURE 3:**
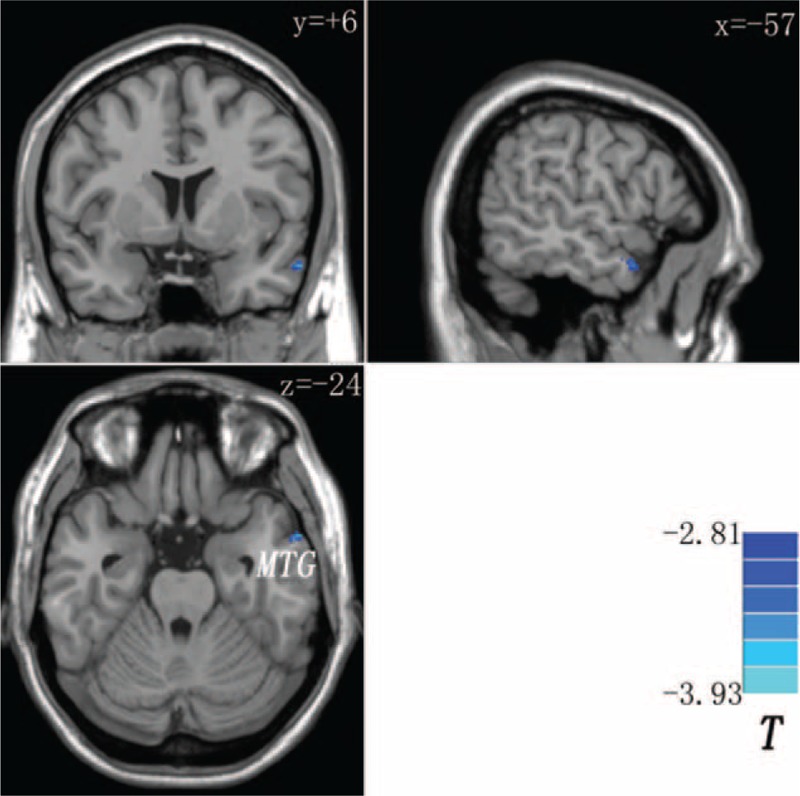
Decreased gray matter volume in the left middle temporal gyrus (MTG) in the combined group (patients and siblings) compared with the controls. The color bar indicates the T values from 2-sample *t*-tests.

To examine the reproducibility of the GCA findings, the patients and the siblings were pooled into a combined group. Then, voxel-wise two-sample *t*-tests were used to compare the anatomical differences between the combined group and the controls. The peak voxel of the cluster with anatomical deficit in the left MTG (Montreal Neurological Institute: −57, 6, −24, Fig. 3) was chosen as a 6-mm-radius sphere seed for GCA. The GCA analysis steps were repeated as mentioned above, and similar results (Table 4) were obtained as the original findings (Table 3).

**TABLE 4:**
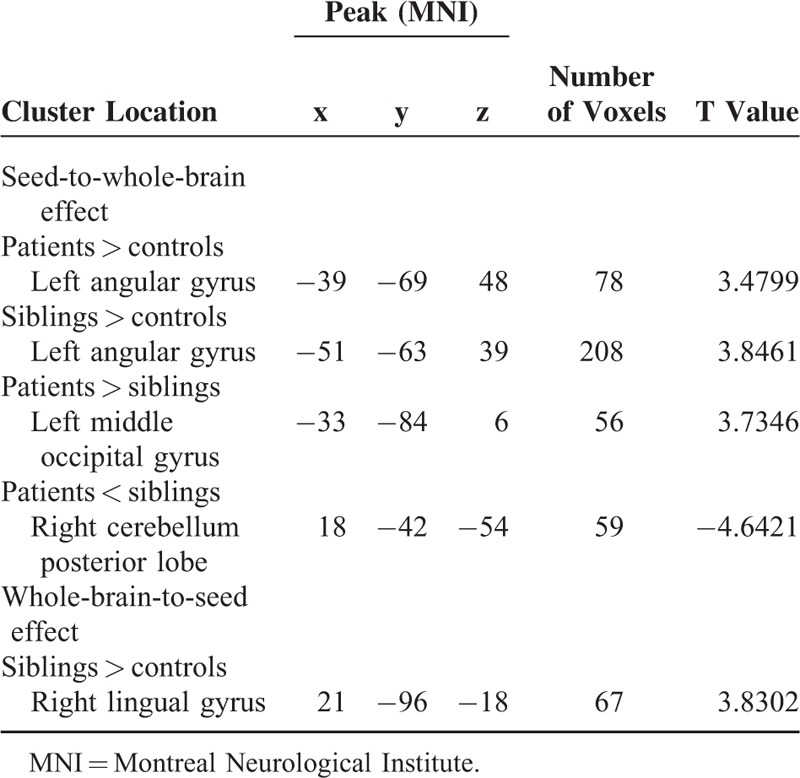
Reproducibility: Regions With Abnormal Causal Effect With the Seed (the Peak Voxel of the Cluster With Anatomical Deficits in the Left Middle Temporal Gyrus)

### Correlations Between Anatomical/Functional Alterations and Clinical Variables in the Patients

**FIGURE 4:**
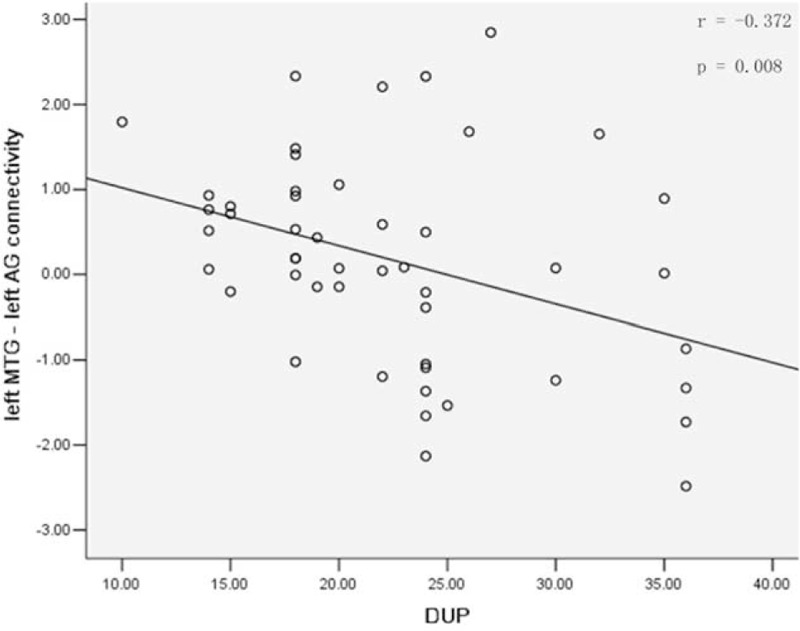
Significantly negative correlation between the z values of the left MTG–left AG causal connectivity and the DUP in the patients (*r* = −0.372, *P* = 0.008). AG = angular gyrus, DUP = duration of untreated psychosis, MTG = middle temporal gyrus.

Significantly negative correlation is observed between the z values of the left MTG – left AG connectivity and the DUP in the patients (*r* = −0.372, *P* = 0.008, Fig. 4). There are no other correlations between anatomical/functional alterations and symptom severity, age, and years of education.

## DISCUSSION

In the present study, we aimed to explore the causal connectivity related to anatomical deficits in schizophrenia and their unaffected siblings, which might serve as potential endophenotypes for schizophrenia. The main findings are that the patients and the siblings share anatomical deficits in the left MTG and increased left MTG–left AG connectivity. Moreover, the left MTG–left AG connectivity negatively correlates to the DUP in the patients.

The present study found that the temporoparietal regions of the CCTCC had excitatory effect related to anatomical deficits in the left MTG in the patients and the siblings. The present findings are in line with the results in schizophrenia patients^43,44^ and unaffected siblings,^45^ reporting increased regional activity and anatomical deficits in the temporoparietal regions. At first glance, increased left MTG–left AG connectivity related to anatomical deficits in the left MTG appears inconsistent with general consideration that the patients have overall decreased connectivity.^46^ The prevailing evidence of overall decreased connectivity in schizophrenia is from studies with chronic and/or medicated patients.^30^ Studies on first-episode, drug-naive patients have found increased frontal connectivity^30,47^ and increased glutamate concentrations.^48,49^ Furthermore, a negative correlation between the left MTG–left AG connectivity and the DUP is observed in the patients. Combined with the findings from unaffected siblings, the present study portrays progressive decrease of the left MTG–left AG connectivity in first-episode, drug-naive schizophrenia with illness duration. The increased connectivity may occur before illness onset as it is seen in the unaffected siblings. According to the concept of endophenotype, the shared anatomical deficits in the left MTG and its increased connectivity with the left AG may be considered as potential endophenotypes for schizophrenia.

Previously, Fair et al^50^ examined the thalamocortical connectivity in healthy children, adolescents, and adults, and observed that the thalamocortical connectivity was present an inverted U-curve with maximal point in healthy adolescents. Since our participants are at the developmental stage from adolescents to adults (aged from 16–30 years), the development of brain connectivity is supposed to be disrupted by schizophrenia, and remains at a relatively high point of the inverted U-curve. Therefore, the present findings provide supporting information to the neurodevelopmental model of schizophrenia.

The increased connectivity is also informative when conceived from its physiological meaning. Increased connectivity is usually regarded as compensatory reallocation or dedifferentiation.^51–56^ We speculated that the increased connectivity in the present study might be the compensatory effort to anatomical deficits in the left MTG, although direct evidence of the compensatory effort is absent. Further evidence is needed to warrant or to refute our speculation.

There are 2 novel aspects in this study. First, we started to identify the anatomical deficits shared by the patients and the siblings. Then, the left MTG with shared anatomical deficits was applied as seed to detect abnormal causal connectivity shared by the patients and the siblings. Previous studies have demonstrated that unaffected siblings share similar brain anatomical and functional abnormalities with the patients.^23,24,26,57–60^ Therefore, the present analysis approach enhances the specificity of identified anatomical and functional abnormalities as potential endophenotypes for schizophrenia.

The second novel aspect is the recruitment of the patients and the siblings unrelated to each other. In this study, only 14 patients and 14 siblings are sib pairs, and the other participants are from different families. The shared genetic background between the patients and the siblings includes susceptibility genes for schizophrenia and genes unrelated to schizophrenia. As mentioned in a previous study,^61^ the recruitment of the patients and the siblings unrelated to one another can reduce the confounding effect from genes unrelated to schizophrenia, and thus enhances the specificity to identify potential endophenotypes for schizophrenia. Furthermore, the patients and the siblings from different families have different early life and environment. The present recruitment approach can also reduce the confounding effect from environmental factors seen in previous sib pair studies^62^ and increases the heritable effect to identify potential endophenotypes.

There are some limitations in the present study. First, neuropsychological tests were not performed in this study, and we cannot examine the correlations between abnormal causal connectivity and neuropsychological parameters. Second, we focused on anatomical and functional abnormalities shared by the patients and the siblings. This approach enhances the specificity to identify potential endophenotypes for schizophrenia. For the same reason, other meaningful results for schizophrenia may be neglected. Finally, this study is conducted at rest, and we can only deduce that abnormal causal connectivity is associated with the general neurobiology in schizophrenia but not with a particular model of task or activity.

Despite the limitations, the present study establishes that anatomical deficits in the left MTG and its increased causal connectivity with the left AG may serve as potential endophenotypes for schizophrenia. The novel analysis method and recruitment approach can enhance the specificity of potential endophenotypes identified for schizophrenia.
